# Rate of malignancy for thyroid nodules with AUS/FLUS cytopathology in a tertiary care center – a retrospective cohort study

**DOI:** 10.1186/s40463-021-00530-0

**Published:** 2021-10-11

**Authors:** Kalpesh Hathi, Tarek Rahmeh, Vicki Munro, Victoria Northrup, Ali Sherazi, Christopher J. Chin

**Affiliations:** 1 Dalhousie Medicine New Brunswick, 711 Millidge Avenue, Suite A, Saint John, NB E2K 2N7 Canada; 2 Department of Laboratory Medicine, Horizon Health Network, Saint John Regional Hospital, Saint John, NB Canada; 3 Department of Medicine, Horizon Health Network, Division of Endocrinology, Saint John Regional Hospital, Saint John, NB Canada; 4 Department of Surgery, Division of Otolaryngology – Head & Neck Surgery, Dalhousie University, Halifax, Nova Scotia Canada

**Keywords:** Thyroid, Bethesda, AUS/FLUS, Nodule, Cytopathology, FNA, Malignancy rate

## Abstract

**Background:**

Thyroid nodules are stratified through fine-needle aspiration (FNA) and are often categorized using The Bethesda System for Reporting Thyroid Cytopathology, which estimates the risk of malignancy for six cytopathological categories. The atypia of undetermined significance (AUS) and follicular lesion of undetermined significance (FLUS) categories have varying malignancy rates reported in the literature which can range from 6 to 72.9%. Due to this heterogeneity, we assessed the malignancy rate and effectiveness of repeat FNA (rFNA) for AUS/FLUS thyroid cytopathology at our institution.

**Methods:**

Electronic health records of patients with AUS/FLUS thyroid cytopathology on FNA at our center since the implementation of the Bethesda System on May 1, 2014–December 31, 2019 were retrospectively reviewed. Patient demographics, treatment pathway, and pathology results were collected. The treatment pathway of the nodules, the rFNA results, and the malignant histopathology results were reported. Malignancy rates were calculated as an upper and lower limit estimate.

**Results:**

This study described 182 AUS/FLUS thyroid nodules from 177 patients. In total, 24 thyroid nodules were deemed malignant upon histopathology, yielding a final malignancy rate of 13.2–25.3%. All of the malignancies were variants of papillary thyroid carcinoma. The malignancy rate of the nodules which underwent resection without rFNA (21.5%) was lower than the malignancy rate of the nodules which underwent resection after rFNA (43.8%). 45.5% of the rFNA results were re-classified into more definitive categories.

**Conclusion:**

The malignancy rate of AUS/FLUS thyroid cytopathology at our center is in line with the risk of malignancy stated by the 2017 Bethesda System. However, our malignancy rate is lower than some other Canadian centers and approximately half of our rFNAs were re-classified, highlighting the importance of establishing center-specific malignancy and rFNA re-classification rates to guide treatment decisions.

## Background

The incidence of thyroid cancer continues to increase in Canada and globally [1–4]. Palpation can detect a thyroid nodule in approximately 4% of the general population [1, 5], however ultrasonography can detect nodules in 19–67.6% of the population [1, 6–8]. Thyroid nodules have been reported in up to 64.6% of autopsies [1, 9]. The more frequent usage of advanced medical imaging has resulted in an increased number of thyroid nodules found incidentally, which is hypothesized to partially account for the increased incidence of thyroid cancer [1–4]. Despite this increased incidence, the prognosis of thyroid cancer remains highly favorable, with an overall five-year survival rate of 98% in Canada [2, 3].

Fine-needle aspiration (FNA) is a safe, accurate and cost-effective procedure used to assess thyroid cytopathology and risk of malignancy (ROM) [10]. The Bethesda System for Reporting Thyroid Cytopathology has reduced ambiguity surrounding thyroid FNA results, subsequently decreasing the rate of unnecessary thyroidectomies [11, 12]. The Bethesda System separates thyroid cytopathology into six categories and for each category, the “usual management” and ROM is provided [11]. The management of the atypia of undetermined significance/follicular lesion of undetermined significance (AUS/FLUS) category has been challenging due to its indeterminant nature and heterogeneous reports of malignancy rates [13, 14]. The original Bethesda System states a ROM of ~ 5–15% for this category, and repeat FNA (rFNA) is recommended [11]. However, various institutions have reported widely ranging malignancy rates from 6 to 72.9% [13–16].

In 2016, histopathology of non-invasive encapsulated follicular variant of papillary thyroid carcinoma (NEFV-PTC) was re-classified as non-invasive follicular thyroid neoplasm with papillary-like nuclear features (NIFTP) to represent its indolent nature [17]. The updated 2017 Bethesda System states a 6–18% ROM for the AUS/FLUS category when ﻿NIFTP is not considered malignant, and a ~ 10–30% ROM when NIFTP is considered malignant [18]. The 2017 Bethesda System recommends rFNA, molecular testing, or lobectomy for AUS/FLUS nodules [18].

Despite this update, there is heterogeneity in malignancy rates amongst Canadian centers and between centers in Canada and the United States [19–22]. Due to this lack of consensus, it is important to determine malignancy rates specific to populations of interest [19, 22, 23].

This study assesses the institution-specific malignancy rate and the effectiveness of rFNA for thyroid nodules with AUS/FLUS cytopathology at the Saint John Regional Hospital in Saint John, New Brunswick, the province’s largest tertiary care centre with a catchment area serving a population of ~ 170,000 [24, 25]. To the best of our knowledge this is the first study outlining AUS/FLUS thyroid cytopathology malignancy rate and treatment in New Brunswick, Canada. The results from our center add to the variable literature surrounding AUS/FLUS thyroid cytopathology and are discussed in relation to reported malignancy rates, specifically in a Canadian context.

## Methods

Approval for retrospective data collection was obtained from the Horizon Health Network Research Ethics Board. All thyroid FNA results since the implementation of the Bethesda System in May 1, 2014–December 31, 2019 were screened, and all patients during this period who were ≥ 18 years old at the time of FNA and had AUS/FLUS thyroid cytopathology were included in the study.

Electronic health records were retrospectively reviewed. The patients’ biological sex, age at the time of first AUS/FLUS cytopathology result, treatment pathway, and pathology results were collected. A nodule was deemed malignant or benign based on surgical histopathology only. Histopathology was correlated to the nodule targeted by FNA by associating the location and size of the nodule. A sub-centimeter microcarcinoma not targeted by the FNA and not found within a larger nodule targeted by FNA was defined as incidental. The presence of an incidental microcarcinoma was noted but deemed benign for the purpose of this study. NIFTP/NEFV-PTC histopathology was considered malignant in this study as the inclusion period pre-dated the 2016 nomenclature and classification adjustment [17].

FNA cytopathology and surgical histopathology were reviewed by in-house pathologists. A specialist in Head and Neck pathology external to our institution was consulted in challenging cases. Final treatment decision making was in collaboration between the patient and the treating physicians. Unless more concerning features were present, AUS/FLUS surgical patients were referred for lobectomy, and if a thyroid carcinoma was identified, contralateral completion thyroidectomy was recommended.

### Data analysis

The proportion of all thyroid FNAs which were classified as AUS/FLUS was calculated. The treatment pathway of the AUS/FLUS thyroid nodules and the distribution of rFNA results were described as percentages. If multiple rFNAs were performed for the same nodule, the most definitive rFNA result was reported. The malignancy rate was calculated for the nodules which underwent immediate resection and those which underwent resection after rFNA. Finally, the distribution of malignant histopathology was calculated.

The final malignancy rate was calculated as an upper limit estimate (ULE) and a lower limit estimate (LLE), as described by Ho et al. [23]. The ULE was the malignancy rate of the resected nodules. The LLE was calculated under the assumption that all the unresected nodules were benign, and therefore was a malignancy rate of all AUS/FLUS cytopathology results.

## Results

During our inclusion period 1130 thyroid FNAs were performed at our center, of which 205 (18.1%) yielded AUS/FLUS cytopathology, 23 of which were rFNA results of previously categorized AUS/FLUS nodules and thus were not included as separate nodules. A total of 182 thyroid nodules with AUS/FLUS cytopathology from 177 patients were included in this study. The patients had a mean age of 58.5 +/− 15.5 years at the time of the AUS/FLUS cytopathology result. The majority of the patients (74.6%, or *n* = 132) were female. 25.4% (*n* = 45) were male.

**Fig. 1:**
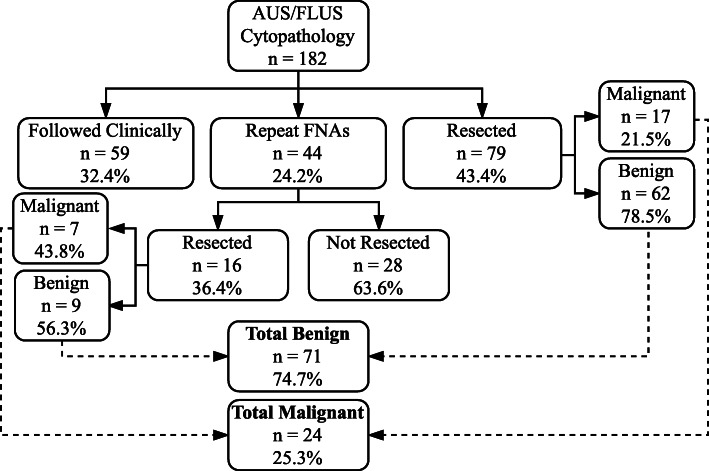
Treatment pathway of the thyroid nodules with AUS/FLUS cytopathology at our center. *Abbreviations*: *FNA –* fine needle aspiration; *AUS/FLUS –* atypia of undetermined significance/follicular lesion of undetermined significance

The treatment pathway of the AUS/FLUS thyroid nodules is described in Fig. 1. Of the 182 nodules, 44 (24.2%) underwent rFNA, 79 (43.4%) underwent resection, and 59 (32.4%) were followed clinically and had no further pathology results available. 16/44 (36.4%) of the nodules which underwent rFNA were resected, and 7/16 (43.8%) of these were malignant. In contrast, 17/79 (21.5%) of the nodules which were resected without rFNA were malignant. The majority of AUS/FLUS surgical patients (84.4%) underwent initial lobectomy.

In total 95/182 (52.2%) of the AUS/FLUS nodules were eventually resected, with 24/95 (25.3%) of these nodules yielding malignant histopathology, representing the ULE malignancy rate. 24/182 (13.2%), represents the LLE malignancy rate.

Table 1 represents the results of the 44 rFNAs, 45.5% were re-classified into more definitive categories, whereas 54.5% remained indeterminant (AUS/FLUS or non-diagnostic). The most common rFNA result was AUS/FLUS (36.4%) and the most common rFNA re-classification result was benign (25.0%).

**Table 1:** Results of the 44 rFNAs after initial AUS/FLUS cytopathology result

Classification	***N*** = (%)	rFNA Result	Frequency (%)	Resected (%^a^)	Malignant (%^b^)
Indeterminant	24 (54.5%)	AUS/FLUS	16 (36.4%)	6 (37.5%)	2 (33.3%)
		Non-Diagnostic	8 (18.2%)	2 (25.0%)	0 (0%)
Re-Classified	20 (45.5%)	Benign	11 (25.0%)	1 (9.1%)	0 (0%)
		FN/SFN	5 (11.4%)	3 (60.0%)	1 (33.3%)
		SFM	4 (9.1%)	4 (100.0%)	4 (100.0%)
		Malignant	0 (0%)	N/A	N/A

*Abbreviations*: *AUS/FLUS* atypia of undetermined significance/follicular lesion of undetermined significance, *FN/SFN* follicular neoplasm/suspicious for follicular neoplasm, *SFM* suspicious for malignancy

^a^Percent of frequency

^b^Percent of resected

Table 2 represents the histopathology reported from the 24 malignant outcomes. All were variants of papillary thyroid carcinoma (PTC). Three (12.5%) were sub-centimeter papillary thyroid microcarcinomas (PTMC) which were either targeted by the FNA or were found within a larger nodule which was targeted by the FNA. Incidental PTMCs were present in 14 (14.7%) of the resected specimens, 5 (5.3%) were found alongside macro-carcinomas in the indexed nodule, and 9 (9.5%) did not have a macro-carcinoma present and were categorized as benign (Table 3).

**Table 2:** Histopathology of 24 malignancies from thyroid nodules with AUS/FLUS cytopathology

Histopathology	Frequency (% of malignant)
FV-PTC	13 (54.2%)
Classic-PTC	4 (16.7%)
PTMC	3 (12.5%)
NIFTP	2 (8.3%)
TCV-PTC	1 (4.2%)
Oncocytic-PTC	1 (4.2%)

*Abbreviations: FV-PTC* follicular variant of papillary thyroid carcinoma, *Classic-PTC* classic or not specified variant of papillary thyroid carcinoma, *PTMC* papillary thyroid microcarcinoma, *NIFTP* non-invasive follicular thyroid neoplasms with papillary-like nuclear features, *TCV-PTC* tall cell variant of papillary thyroid carcinoma, *Oncocytic-PTC* oncocytic variant of papillary thyroid carcinoma

**Table 3:** Description of 14 incidental microcarcinomas

Histopathology	Frequency (% of resected)
With Macro-Carcinoma (Malignant)	5 (5.3%)
Without Macro-Carcinoma (Benign)	9 (9.5%)
Total	14 (14.7%)

## Discussion

The Bethesda System for Reporting Thyroid Cytopathology effectively standardizes categorization of thyroid FNA results [12, 18]. However, the indeterminate nature of AUS/FLUS cytopathology has resulted in heterogeneous reports of malignancy rates for this category [13, 14]. The malignancy rate of AUS/FLUS thyroid cytopathology at our center is 13.2–25.3%.

Many centers report malignancy rates above the ~ 5–15% ROM stated by the 2009 Bethesda System (Table 4) [11, 13, 14, 16, 19–23, 26–33]. The 2017 Bethesda System states a ~ 10–30% ROM for AUS/FLUS thyroid cytopathology [18] which coincides well with our malignancy rate (13.2–25.3%). However, centers do report malignancy rates exceeding 30% (Table 4) [16, 19–21, 33].

**Table 4:** Malignancy rate of AUS/FLUS thyroid cytopathology reported at various centers

Series	Country	Period	AUS/FLUS Rate %	LLE Malignancy Rate %	ULE Malignancy Rate %
Present Study	Canada	2014–19	18.1 (205/1130)	13.2 (24/182)	25.3% (24/95)
The Bethesda System [11]	N/A	2009	≤7	~ 5–15	N/A
The Bethesda System [18]	N/A	2017	≤10	~ 10–30	N/A
Bernstein et al. [19]	Canada	2010–13	12.0	37 (86/233)	46 (86/187)
Erivwo & Ghosh [20]	Canada	2010–13	5.5	29.8 (54/181)	N/A
Oosthuizen et al. [21]	Canada	2010–16	N/A	N/A	32 (30/93)
Williams et al. [22]	Canada	2006–10	18.8	N/A	24.7 (N/A)
Wu et al. [15]	United States	2006–08	27.2	N/A	6 (3/51)
Hong et al. [16]	South Korea	2011–14	N/A	10.2 (70/687)	72.9 (70/96)
Ho et al. [23]	United States	2008–11	8.0	26.6 (144/541)	37.8 (144/381)
Cavalheiro et al. [26]	Brazil	2010–14	N/A	N/A	15.7 (75/478)
Mileva et al. [27]	Republic of Macedonia	2012–16	5.9	N/A	31.2 (35/112)^a^
Al-Abbadi et al. [28]	Saudi-Arabia	2010–14	4.4	N/A	29 (12/42)^a^
Wong et al. [29]	United States	2008–12	9	N/A	33.1 (60/181)
Yaprak Bayrak et al. [30]	Turkey	2012–17	4.2	N/A	25.0 (27/108)
Sullivan et al. [31]	United States	2003–12	6	17 (56/332)	33 (56/168)
Topaloglu et al. [32]	Turkey	N/A	N/A	N/A	23.4 (105/449)
Vanderlaan et al. [33]	United States	2005–09	10.9	29.0 (96/331)^a^	48.2 (96/199)

*Abbreviations: AUS/FLUS* atypia of undetermined significance/follicular lesion of undetermined significance*, LLE* lower limit estimate*, ULE* upper limit estimate

^a^Includes malignant/benign diagnosis based on histopathology, core needle biopsy, or rFNA

In terms of Canadian data, the malignancy rate seems to vary geographically. The largest Canadian series of AUS/FLUS thyroid cytopathology was conducted in an Ontario center and yielded a 37–46% malignancy rate [19], which is noticeably higher than our center. A study in Newfoundland also reported a higher LLE malignancy rate of 29.8% [20]. However, centers in Western Canada [21] and Nova Scotia [22] reported ULE malignancy rates of 32 and 24.7% respectively, which are similar to our study. Overall, our malignancy rate is within the lower end of those reported in the literature (Table 4).

These higher malignancy rates may suggest the need for further adjustment of the ROM stated in the 2017 Bethesda System. However, it is also possible that there is no generalizable ROM for this cytopathology [34]. Rather, center and population specific factors may account for the variability of malignancy rates [19, 22, 23]. For example, Ho et al., [23] noted their higher malignancy rate (26.6–37.8%) may be attributed to their high volume cancer center setting, potentially resulting in a referral bias of more concerning nodules. Conversely, Cavalheiro et al. [26] reported a 15.7% malignancy rate from a center where all AUS/FLUS thyroid nodules were surgically resected and Wu et al. [15] reported a 6% malignancy rate for FNAs from community practice settings. As shown in these studies, there is a wide variability in the malignancy rate for this cytological diagnosis, and this illustrates the importance and value of identifying the malignancy rate at ones’ own institution.

Our center had a high rate of AUS/FLUS diagnosis (18.1%) compared to the Bethesda recommendation of ≤10% [18]. The common “overuse” of the category and the greater inter−/intra-observer variability of AUS/FLUS diagnosis has been noted [13]. The other Canadian center with a similar malignancy rate to this study also reported a high AUS/FLUS rate (18.8%) [22], whereas the two Canadian centers with higher malignancy rates had lower AUS/FLUS rates (5.5 and 12.0% respectively) [19, 20]. This relationship is observed in many studies cited in Table 4, and it is likely that lower AUS/FLUS rates correspond to higher malignancy rates. It has been hypothesized that higher AUS/FLUS rates (> 15%) result in more benign or non-diagnostic cases categorized as AUS/FLUS, whereas lower rates (< 5%) are associated with reduced sensitivity [13, 35], which is important knowledge for clinical decision making.

The Bethesda System recommends lobectomy, rFNA, or molecular testing for AUS/FLUS cytopathology [18]. At our center, the malignancy rate of nodules resected after rFNA (43.8%) was noticeably higher than those proceeding directly to surgery (21.5%). This can likely be attributed to nodules re-classified in more concerning categories (FN/SFN and SFM) representing 43.8% of the resections after rFNA. In contrast, the majority of nodules re-classified by rFNA yielded benign cytopathology (25.0%) and only one was resected. This is in line with previous studies [31, 36], and demonstrates the potential for rFNA to facilitate management of nodules originally classified as AUS/FLUS. However, varying rFNA re-classification rates are reported amongst Canadian centers [36, 37], suggesting value in determining the institutional rFNA re-classification rates.

This study has some limitations. Due to its retrospective nature, 52.2% of the thyroid nodules with AUS/FLUS cytopathology underwent surgical resection, while the remaining nodules lacked histopathology results and were not definitively classified as benign or malignant. They were assumed to be benign when calculating the LLE malignancy rate, however it is possible they contained an indolent, malignant neoplasm. Also, the number of rFNAs which underwent resection is small and a larger sample size would be required to provide stronger conclusions for this sub-group. Finally, this study considered NIFTP/NEFV-PTC histopathology as malignant due to the inclusion period pre-dating our institution’s implementation of the 2016 nomenclature change [17]. A future study comparing the malignancy rate pre and post implementation of NIFTP nomenclature at our center would be of interest.

## Conclusion

The malignancy rate of thyroid nodules with AUS/FLUS cytopathology at our center is 13.2–25.3%. Our malignancy rate is in line with the ROM stated by the Bethesda System (~ 10–30%) [18]. Repeat FNA was helpful in re-classifying nearly 50% of the nodules that were biopsied and remains a useful technique in triaging these nodules. This study highlights the importance of establishing center-specific malignancy and rFNA re-classification rates to guide treatment decisions.

## Data Availability

The datasets used and analysed during the current study are available from the corresponding author upon reasonable request and approval from the Horizon Health Network Privacy Officer.

## Abbreviations

FNA: Fine-needle aspiration
ROM: Risk of malignancy
AUS: Atypia of undetermined significance
FLUS: Follicular lesion of undetermined significance
rFNA: Repeat fine-needle aspiration
NEFV-PTC: Non-invasive encapsulated follicular variants of papillary thyroid carcinoma
NIFTP: Non-invasive follicular thyroid neoplasms with papillary-like nuclear features
ULE: Upper limit estimate
LLE: Lower limit estimate
FN: Follicular neoplasm
SFN: Suspicious for a follicular neoplasm
SFM: Suspicious for malignancy
PTC: Papillary thyroid carcinoma
PTMC: Papillary thyroid microcarcinoma
FV-PTC: Follicular variant of papillary thyroid carcinoma
Classic-PTC: Classic or not otherwise specified variant of papillary thyroid carcinoma
TCV-PTC: Tall cell variant of papillary thyroid carcinoma
Oncocytic-PTC: Oncocytic variant of papillary thyroid carcinoma
